# Anthropogenic tritium as a continental-scale tracer in river-derived recharge

**DOI:** 10.1038/s44221-026-00616-x

**Published:** 2026-03-23

**Authors:** Jared van Rooyen, Torsten Vennemann, Roland Purtschert, Annette Affolter Kast, Matthias S. Brennwald, Rolf Kipfer, Oliver S. Schilling

**Affiliations:** 1 Water Resources and Drinking Water, Eawag, Swiss Federal Institute of Aquatic Science and Technology, Dübendorf, Switzerland; 2 Hydrogeology, Department of Environmental Sciences, University of Basel, Basel, Switzerland; 3 Institute of Earth Surface Dynamics, University of Lausanne, Lausanne, Switzerland; 4 Climate and Environmental Physics and Oeschger Center for Climate Change Research, University of Bern, Bern, Switzerland; 5 Applied and Environmental Geology, Hydrogeology, Department of Environmental Sciences, University of Basel, Basel, Switzerland; 6 Institute of Biogeochemistry and Pollutant Dynamics and Institute of Geochemistry and Petrology, Swiss Federal Institute of Technology Zurich (ETHZ), Zurich, Switzerland

**Keywords:** Hydrology, Environmental monitoring

## Abstract

Groundwater, enhanced through managed aquifer recharge (MAR), plays a central role in mitigating current and future water stress. Here we evaluate anthropogenic and natural water isotopes as tracers of groundwater flow dynamics within alluvial MAR systems. High-resolution sampling (daily/weekly) of stable isotopes (δ^18^O and δ^2^H) and tritium (^3^H), influenced by nuclear power plant effluents, is used to trace and quantify the movement of infiltrated river water through an alluvial aquifer along the Rhine River in Switzerland. Time-series deconvolution is applied to quantify the tracer-based travel time distribution and to predict travel times throughout the entire MAR scheme. The results demonstrate the suitability of ^3^H as a quasi-conservative travel time tracer in systems where the infiltrating river water is marked by nuclear power plant discharges—a situation prevalent along the banks of many large river basins globally. Deuterium excess proved equally effective as a bulk travel time tracer, reflecting distinct seasonal meltwater signals expected in major European rivers. These findings quantify MAR recovery rates and wellhead protection zones, supporting sustainable groundwater management under natural and anthropogenic pressures.

## Abstract (web summary)

Groundwater, enhanced through managed aquifer recharge, is crucial for alleviating water stress. This study demonstrates that isotopic tracers, including tritium from nuclear power plant effluents, can be used to map groundwater flow in Swiss alluvial systems, revealing insights into groundwater travel time distributions and informing sustainable groundwater management globally.

## Main

Water resource management is fundamental to sustainable development but must increasingly contend with intensifying pressures from climate variability, land-use change and competing human demands^1,2^. A clear shift in water management and policy is the increasing dependence on groundwater resources, underpinned by constant pressure from growing food and energy demands^3,4^. Although this shift is often framed as a risk and assessed as a degree of vulnerability to depletion or a deterioration in quality^5–7^, industry and agriculture identifies groundwater as a potential for stability and growth, a resource that can be exploited indefinitely for potable supply, geothermal energy or irrigation^8,9^. The ability to quantify the extent of seasonal waters and the potential of groundwater resources in strategic potable water systems is central also to the United Nations Sustainable Development Goals^10^, environmental protection efforts and renaturalization of river- and groundwater-dependent ecosystems.

A key obstacle to this understanding is the invisible nature of groundwater. Unlike surface water bodies, changes in aquifer conditions are largely unobservable without dedicated monitoring or modelling. This uncertainty is exacerbated by climate change, which alters climate patterns, perturbing interception and recharge rates and shifts hydrological cycles and dependence in ways that are difficult to predict^11–15^. In response, many water-stressed regions have adopted managed aquifer recharge (MAR) strategies, including induced bank filtration, surface spreading and well injection, to stabilize supply^16–19^. Yet, these interventions often proceed with limited information about their long-term hydraulic or geochemical consequences^3^. To address these challenges, recent tracer-based studies offer a path forward, enabling quantitative assessment of groundwater transit times and mixing processes over operationally relevant scales in MAR systems^20–26^, although dependent on a thorough understanding of subsurface residence times and flow paths^27–30^.

Isotopes of the water molecule, such as ^18^O and ^2^H, are particularly effective in delineating recharge sources and mixing processes owing to their conservative behaviour in the hydrological cycle^31–33^. In addition, transient tracers such as tritium, chlorofluorocarbons and sulfur hexafluoride have been used to estimate groundwater residence times and assess modern recharge contributions^34–38^. These techniques, when applied in combination with hydrogeological models, have enabled researchers to track groundwater movement at high resolution, offering an empirical basis for assessing aquifer sustainability^39–41^. However, these approaches also have limitations, particularly in complex systems where mixing, dispersion and variable recharge rates introduce uncertainty in age-dating models and tracer interpretations^42,43^, for example, when detection limits exceed tracer signals^26^ or variability is too high for end-member identification^44^. To enhance the accuracy of tracer-based assessments in MAR settings, techniques have been extended from event-based tests to longer-term, continuous time-series methods, which enable the reconstruction of travel times and flow distributions in a much more detailed manner^45–48^. These recent techniques provide critical insight into the efficiency of MAR operations, enabling water managers to optimize recharge and abstraction and assess the sustainability of induced infiltration.

**Fig. 1:**
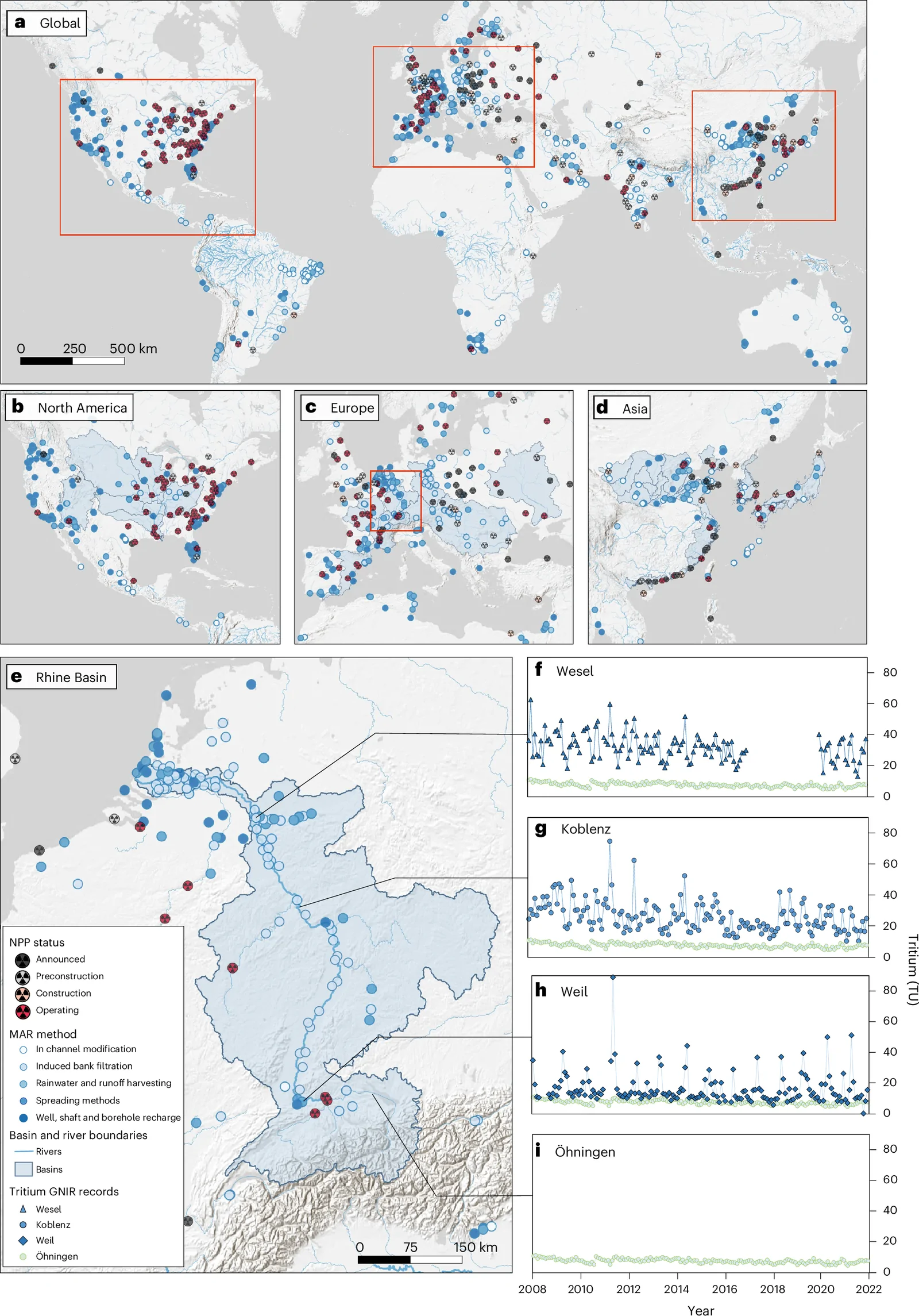
NPPs and MAR in major river basins. **a**–**d**, Global map (**a**) showing spatial overlap between operating, under-construction and proposed NPPs^76^ and different MAR system types^77^. MAR methods include in-channel modifications, induced bank filtration, runoff harvesting, surface spreading and well/borehole recharge. Major river basins with colocated NPPs and MAR are highlighted. Insets highlight regional detailed maps for North America (**b**), Europe (**c**) and Asia (**d**). **e**, Locations of NPPs and MAR sites across the Rhine Basin (highlighted blue), with the study area located in the southern headwaters of the Rhine basin. **f**–**i**, The 25-year tritium concentration records at the major Rhine gauging stations are presented across the Basin, namely, Wesel (**f**), Koblenz (**g**), Weil (**h**) and Öhningen (**i**)^78^. The natural background concentration in the Rhine as represented by the NPP unaffected gauging station in Öhningen is shown in **f**–**i** for comparison. GNIR, Global Network of Isotopes in Rivers. For the station Wesel, additional data inside the temporal gap of the WISER dataset have been presented in ref. ^79^. Basemap tiles in **a**–**e** from CartoDB under a Creative Commons license CC BY-SA 2.0 with map data from OpenStreetMap under an Open Data Commons Open Database License (https://opendatacommons.org/licenses/odbl).

The manipulation of hydrological systems, for example, through MAR, is a key component of climate change adaption^14,49^. Similarly, despite its risks, the production of energy through nuclear power plants (NPPs) represents a pathway to more sustainable industry, with 170 (419 cores) operational power plants distributed globally (Fig. 1). The distribution of active and planned NPPs across major river basins, which produce large volumes of nuclear release waters, provides a unique opportunity to assess the efficacy of NPP-released radiogenic tracers as indicators of river waters in proximal groundwater systems and recharge schemes on continental scales^50–52^. This distribution reveals that at least 26 major river basins worldwide have the spatial and hydrological configuration to support the use of NPP-derived tritium as an environmental tracer for MAR performance assessment. Within these basins, MAR infrastructure serves communities where an estimated 320 million people rely directly or indirectly on MAR-sourced water for drinking, irrigation and food production. Europe’s Danube and Rhine basins stand out as high-potential testbeds for the method, combining dense reactor networks, well-monitored hydrology and extensive MAR coverage. Other strong candidates include the Elbe, Ganges, La Plata and Amu Darya–Syr Darya basins, as well as select sites in North America, East Asia and East Africa. Across these settings, tracer applicability depends on upstream NPP positioning relative to MAR systems, sufficient proximity to limit dilution, hydrological continuity between release and abstraction points, and stable isotope backgrounds for signal contrast. Together, these factors define the regions where long-term monitoring could yield insights into MAR performance under operational conditions.

Here, we explore the suitability of isotope tracers, both stable (δ^18^O, δ^2^H and deuterium excess (d-excess)) and radiogenic (^3^H), to quantify the travel times of managed recharge in one of Europe’s largest MAR schemes along the Rhine River in Basel, Switzerland. Through a combination of high-resolution sampling that covered both seasonal (natural background) and NPP release signals, the methodological framework’s success lies in the integration of these high-resolution time series with non-parametric deconvolution of the seasonally distinct tracer input signals, providing complementary insights into the timing and distribution of travel times from the infiltration to the abstraction points. In addition, interrogation of the spatial distribution of ^3^H as NPP release waters propagate through the MAR system allows us to address the composite nature of groundwater travel time distributions within the system.

## Results

### Observing NPP release waters in major rivers and MAR schemes

The stable water isotope signatures observable in the Rhine River near Basel are generated through the mixing of different waters derived from precipitation that falls throughout the northern half of Switzerland, including the higher-altitude alpine regions. The water is isotopically marked by location, altitude and seasonality of precipitation events and further impacted by distinct snowmelt periods, consistent but seasonally variable baseflow groundwater contributions, and release waters from the large Bodensee (Lake Constance)^53^. With respect to the radioactive isotope ^3^H, which is bound within the water molecule, there is a small natural annual variation in the Rhine River (6–8 tritium units, TU; Fig. 1, Öhningen), yet the variability of ^3^H is impacted by the release of nuclear power cooling waters, which can drastically increase the abundance of ^3^H (>30–60 TU; Fig. 1, Weil). Upstream of the Rhine in Basel, three operational NPPs release cooling waters to its drainage area: Leibstadt (located along the Rhine 50 km upriver), Beznau (located along the Aare River in the canton of Aargau) and Gösgen-Däniken (located along the Aare River in the canton of Solothurn). These releases create distinguishable ^3^H signals in the Rhine waters downstream of release sites, not only in Basel but traceable along the Rhine across the continent until the river reaches the Atlantic Ocean (Fig. 1).

**Fig. 2:**
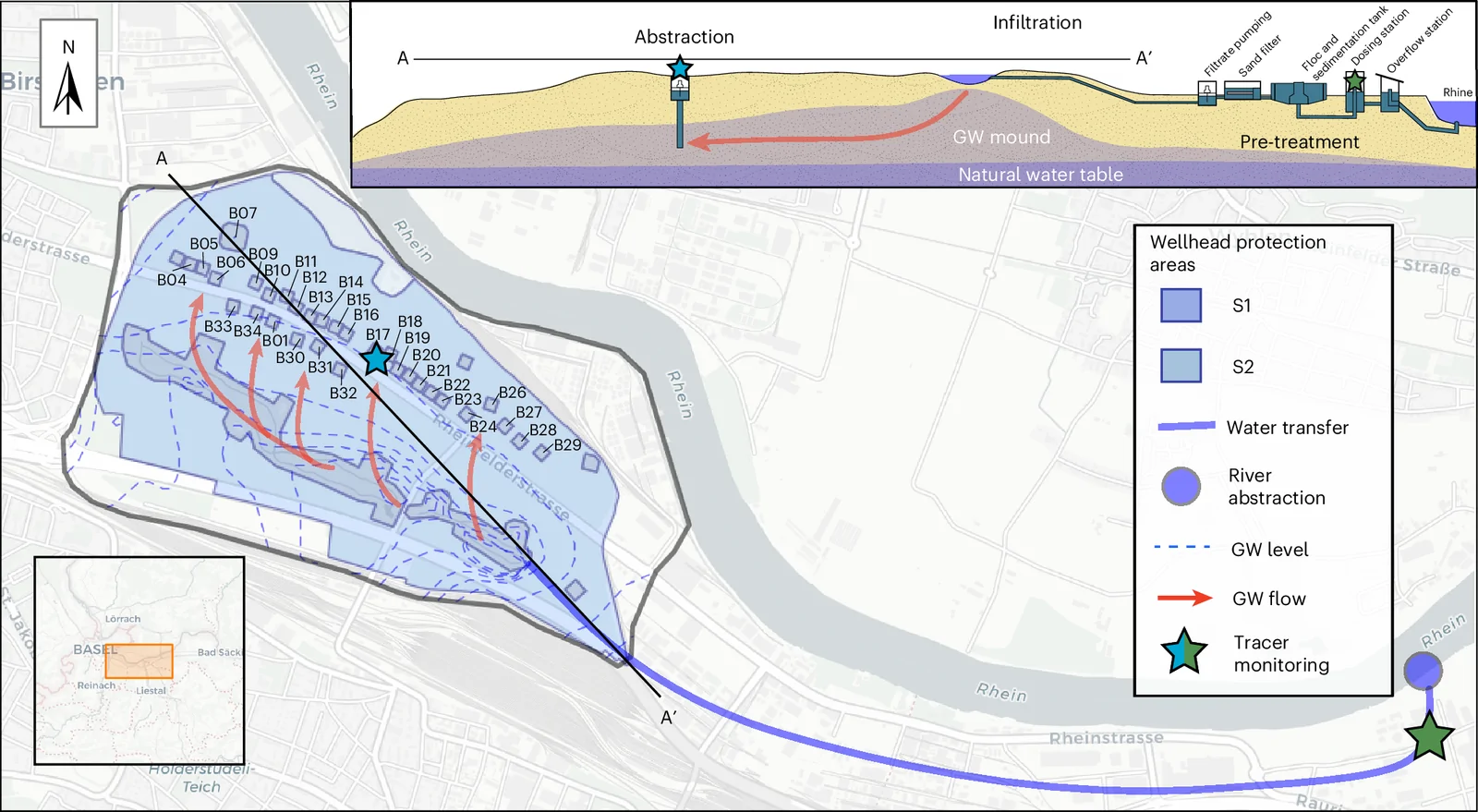
The Hardwald MAR site near Basel, Switzerland. Map of the Hardwald MAR site set on the banks of the Rhine, near Basel, Switzerland. Shown are the Swiss regulatory^65^ wellhead protection zones S1, which delineate the direct surroundings of the abstraction wells and the infiltration channels/ponds, and S2, which encompasses the entirety of the MAR scheme, as well as the transfer line that transports water from the Rhine abstraction point through a pretreatment station and into the infiltration channels and ponds. Environmental isotope tracer measurement locations are shown by star markers (green, infiltrate waters; blue, abstraction waters). Groundwater (GW) levels and generalized flow paths are represented by blue dashed lines and red arrows, respectively. Basemap tiles from CartoDB under a Creative Commons license CC BY-SA 2.0 with map data from OpenStreetMap under an Open Data Commons Open Database License (https://opendatacommons.org/licenses/odbl).

The Hardwald MAR system, which is the largest MAR scheme in the upper Rhine River basin, is located just 5 km upstream of the city of Basel in Switzerland (Fig. 2). The system plays a crucial role in the region’s drinking water production, yielding approximately 15 million m^3^ annually. The configuration of the MAR scheme is designed specifically to pretreat fresh waters from the Rhine, where it undergoes aeration and sand filtration before being directed into surface spreading channels and ponds (Fig. 2). The area, located just downstream of the site where Europe’s largest historic river pollution disaster—the 1986 Sandoz spill—originated, is also surrounded by substantial urban and industrial development as well as legacy landfill sites, all of which have the potential to negatively affect local groundwater quality^54^. The MAR system has therefore been designed to recharge twice as much groundwater as is extracted, creating a ‘groundwater mound’ that safeguards the area from surrounding industrial zones and contaminated sites^54^ (Fig. 2).

### Stable isotope and tritium patterns in infiltrated and abstracted waters

**Fig. 3:**
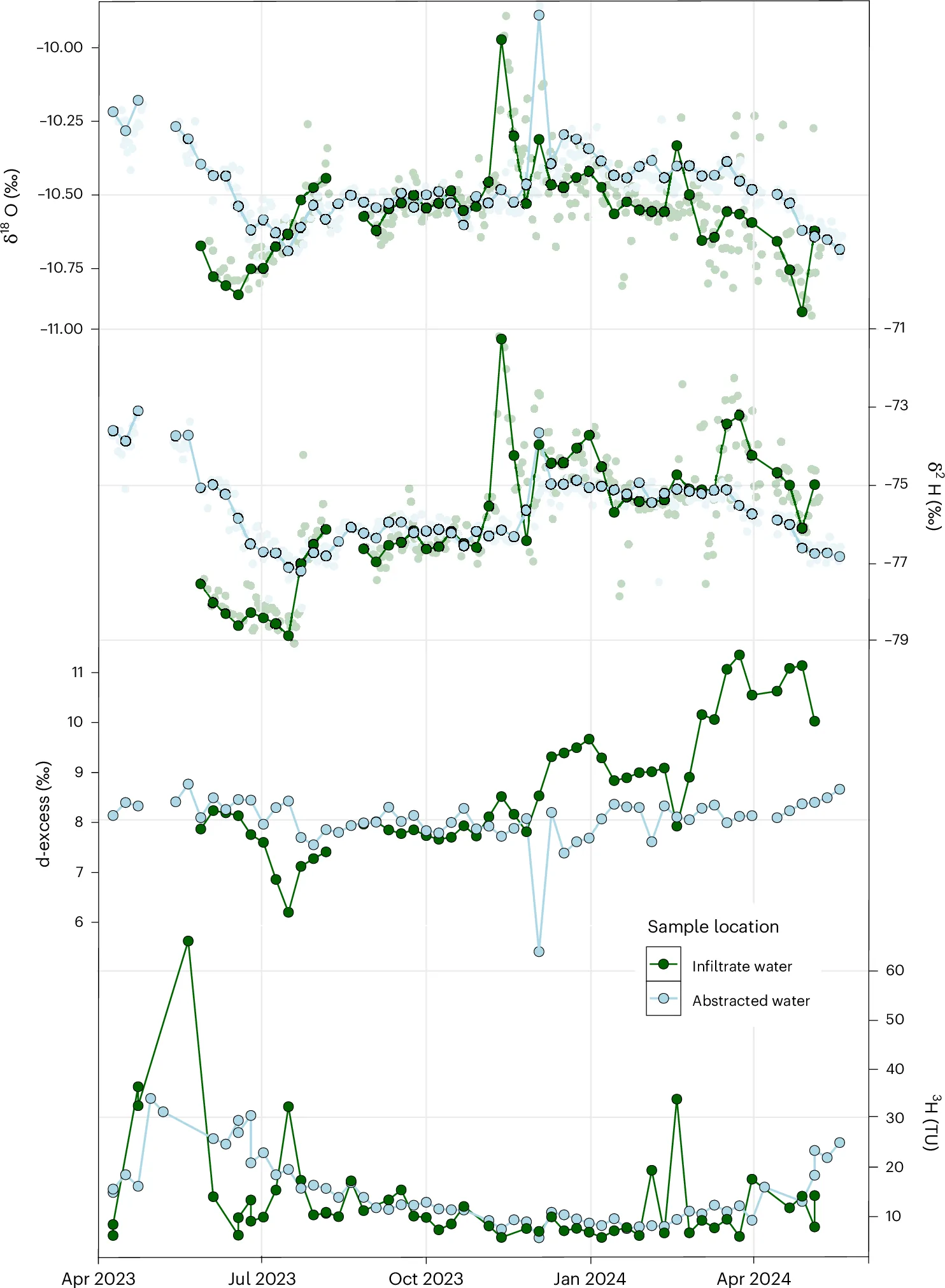
Time series of measured isotopic values of infiltrate and abstracted water. Time series of tracers (δ^18^O, δD, d-excess and ^3^H) analysed as daily (daily stable isotope data are displayed by light circles, weekly data by dark circles, *n* = 356) and weekly (^3^H, *n* = 50) composite samples collected from both the Rhine infiltrate waters (green) and abstracted groundwater (blue) from April 2023 until early June 2024.

The time-series analysis of δ^18^O and δ^2^H from both infiltrated (Rhine water, *n* = 342) and abstracted waters (production borehole B17, *n* = 404) sampled daily throughout 1 year reveals distinct seasonal and temporal variations (Fig. 3). Rhine water δ^18^O fluctuates between −11.35‰and −9.39‰ ±0.05, while groundwater δ^18^O is less variable with a smaller range (between −10.74‰ and −9.75‰ ±0.05). Similarly, δ^2^H varies between −82.9‰ and −69.3‰ ±0.3 in the infiltrated water, while groundwater exhibits a similar lower variability (between −78.4‰ and −73‰ ±0.3). The complete attenuation of highs and lows in isotopic composition, from infiltration to abstraction, reveals that dispersion and mixing along the flow path results in a total loss of identifiable peaks in the δ^18^O and δ^2^H time series, consistent with expectations in porous media and MAR schemes^55^. Hence, while a clear seasonal pattern is evident in δ^18^O and δ^2^H in infiltrated water—with enriched signals in summer and depleted signals in winter—these signals are attenuated too strongly in unconsolidated alluvial sediments to allow tracing of recharge along typical MAR schemes.

Deuterium excess is a derivative of the original δ^2^H and δ^18^O input signals (expressed as δ^2^H − 8·δ^18^O) and a reputable metric for water mass origin^56^. Relatively higher d-excess values in Rhine water (>8‰) suggest predominant influences from source precipitation over evaporative loss (Fig. 3). This phenomenon is particularly clear in the presence of snowmelt in the system in the early summer months^15^, where increased d-excess corresponds to the aridity associated with the air column in which high altitude snow typically falls. Although this distinct change in d-excess values is also attenuated along the flow path, distinct peaks in d-excess values of the infiltrate can be observed in the abstracted water. Hence, in contrast to the δ^2^H and δ^18^O signals, the differences in water mass origin do provide a viable application of stable isotopes as tracers in MAR systems along rivers fed by large snowmelt events.

^3^H concentrations of weekly composite samples taken over the course of 1 year display a clear distinction between the infiltrated (*n* = 52) and abstracted water (*n* = 57), fluctuating around 10 TU (±5 TU) in the infiltrate while showing a generally lower and more stable pattern in groundwater (Fig. 3). There are multiple distinct spikes in ^3^H in infiltrated Rhine water (for example, >60 TU in April 2023 and >35 TU in March 2024), marking the seasonal presence of NPP release waters and forming a strong and distinct signal in the infiltrated water. ^3^H peaks in the abstracted water are delayed and attenuated (33.5 TU maximum), yet not necessarily indicating that recharge water has undergone sufficient radioactive decay to be discernible from ambient levels, implying recent infiltration and thus travel times of a few weeks. The resulting U-shaped pattern in abstracted ^3^H concentrations reflects the convolution of temporally variable NPP release signals with the system’s residence time distribution, rather than dominance of radioactive decay or seasonality in atmospheric background ^3^H concentrations. It arises from the combined effects of temporal variability high NPP ^3^H inputs, the dispersion and mixing of enriched infiltrate waters (supported by the δ^18^O and δ^2^H record) and the persistence of NPP-produced ^3^H in abstracted waters during periods where NPP waters are not present in the infiltrated water.

### The travel time distribution of MAR waters identified by isotope time-series deconvolution

The clear seasonal trends in d-excess and ^3^H data for both infiltrated and abstracted waters provide a basis for computing the observable time delay between shorter-term highs and lows in tracer abundance in the infiltrated and their manifestation in abstracted water. Peak matching estimates travel time by identifying peaks in the tracer concentration time series at the infiltration and abstraction points, computing the time delay between them, yet the computation has no dimension of amplitude and does not consider dispersion or mixing along the groundwater flow path^57–59^. Peak matching applied to the tracer signals of the Hardwald MAR indicates very short first arrival times of 8.3 days for d-excess and ^3^H. These peak matching-based travel times are consistent with first arrivals of previous dye injection tests, which indicated first arrival between 5 and 7 days under a more forced pumping regime but peak/mean arrival between 12 and 28 days, dependent on the location of dye injection^60^.

**Fig. 4:**
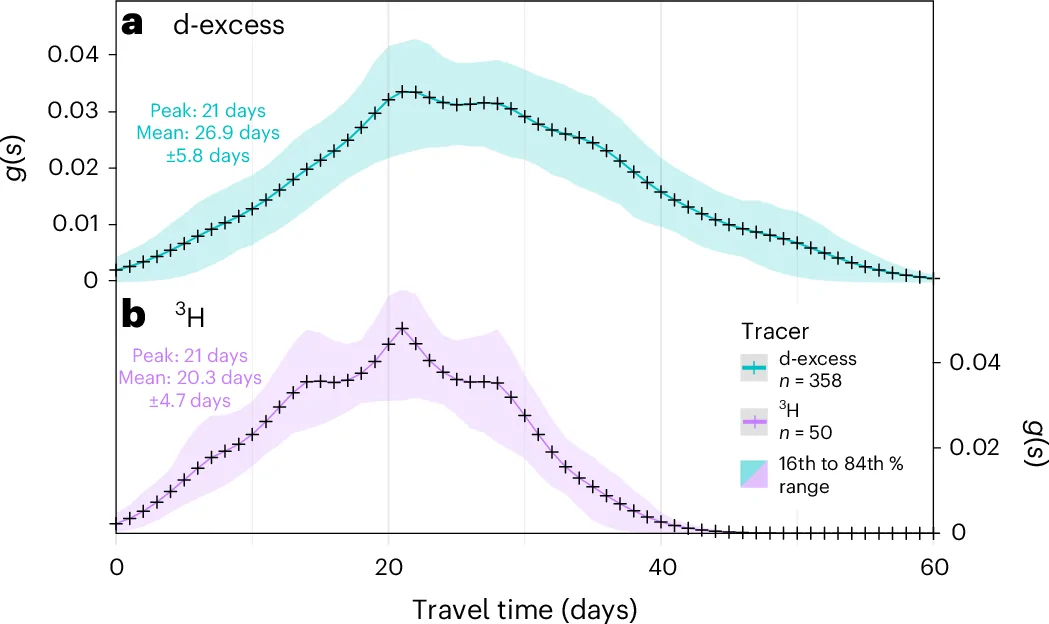
Transfer functions derived for d-excess and ^3^H using non-parametric deconvolution. **a**,**b**, Breakthrough curves for d-excess (**a**) and ^3^H (**b**) were derived from non-parametric deconvolution. The transfer function *g*(*s*) represents the estimated residence time distribution. The solid line denotes the mean of conditional realizations. The shaded area represents the 16th to 84th percentile range of the conditional statistical distributions. The black crosses represent the best non-negative estimate of the breakthrough function at each time step, corresponding to the most probable travel time distribution derived from the deconvolution approach. The time axis is normalized to the first observed breakthrough arrival, ensuring comparability across different tracers.

While peak matching is limited and resolves only clearly observable discrete peaks in measured time series, deconvolution provides a continuous probability distribution of travel times, offering insights into the broader hydrological dynamics including slower pathways and mixing processes^45,46,61^. Time-series non-parametric deconvolution hence detects significantly longer travel times of 21.4 and 21 days for d-excess and ^3^H, respectively, for peak arrivals and 26.9 and 20.3 days for mean arrivals (the point at which half of the input signal has plausibly arrived at the abstraction well) (Fig. 4). This highlights that, while some water moves quickly through preferential pathways (as seen in peak matching), the bulk of abstracted groundwater takes a longer, more dispersed/mixed route before reaching the abstraction well. In addition, both the deconvoluted d-excess and ^3^H signals showed almost complete recovery rates and were able to resolve the input functions without loss, suggesting that all of the abstracted waters originate from the infiltration zones.

The clear advantage of deconvolution over peak matching is its ability to resolve a continuous probability distribution of travel times, thereby capturing slower groundwater pathways and mixing dynamics that discrete peak analyses cannot detect^45,46,61^. The longer tailings (or truncation time) of the travel time transfer functions of d-excess and ^3^H indicate that a large portion of the infiltrated water can remain in the system for weeks to months (36.7–58.8 days). If the measurements are, furthermore, binned into multiday averages of different sizes, one can identify ideal sampling frequency or aggregation duration, which in the present case exposes 5–9 days as the lowest sampling frequency to still produce meaningful results (Supplementary Fig. 1). This optimal sampling frequency is a function of the range of travel times observable in the studied system (from about 1 week up to several months) and the fact that ^3^H and d-excess exhibit clear seasonal fluctuations, which makes them ideally suited for resolving travel times on the order of weeks to months^62–64^. The similar shapes of the travel time transfer functions of ^3^H and d-excess provide a very interesting new perspective on the usefulness of stable water isotopes as tracers for groundwater with longer travel times, as generally it is assumed that ^3^H contains more information about groundwaters with longer travel times than stable isotopes^62–64^. In the studied system, we find that d-excess is an equivalent indicator to ^3^H owing to the distinct shifts in water mass origins within an annual period.

The mixing ratios, computed as the residuals of the peak matching correlation coefficients, show that the abstraction water is almost entirely composed of the artificially infiltrated Rhine water, with fractions ranging from 0.93 (d-excess) to 1.00 (^3^H), supported further by the associated recovery rates from the deconvolution, which ranged between 97% and 99% (Supplementary Table 1). The slightly lower d-excess-based value (0.93) could indicate some influence of mixing with a regional groundwater component. This discrepancy between the ^3^H- and d-excess-based fractions suggests that d-excess may be a particularly useful tracer for identifying different water components in such MAR settings, as d-excess is an indicator of water mass origin and varies systematically within the seasonality. While ^3^H from NPP releases manifests as an excellent tracer for the travel times of the bulk of the water that moves through the system, the fact that it decays and thus is not conservative over longer residence times, makes ^3^H not an ideal tracer for the detection of older water components in which most tritium has already decayed. Yet, the vast majority of modern MAR techniques would not exceed the quasi-conservative timescale of an NPP ^3^H release signal in infiltrate waters, as they are designed to produce travel times that are sufficient for natural filtration and short-term storage, rather than unfeasibly long transit periods.

Systems with morphologies and forcings that differ from the Hardwald MAR scheme, which is characterized by constant infiltration and abstraction rates, are expected to yield systematically different deconvolution responses. Specifically, (1) higher hydraulic gradients would result in narrower transfer function distributions, (2) structural heterogeneity would introduce skewness reflecting the coexistence of fast and slow flow pathways, and (3) spatially distributed infiltration zones would modify the apparent truncation times of the inferred transfer functions. More generally, the ability of deconvolution methods to resolve transient groundwater recharge, flow and discharge processes is constrained by the distinctness of the input signals, the persistence of tracers along flow paths, and the extent of mixing and dispersion within the system.

**Fig. 5:**
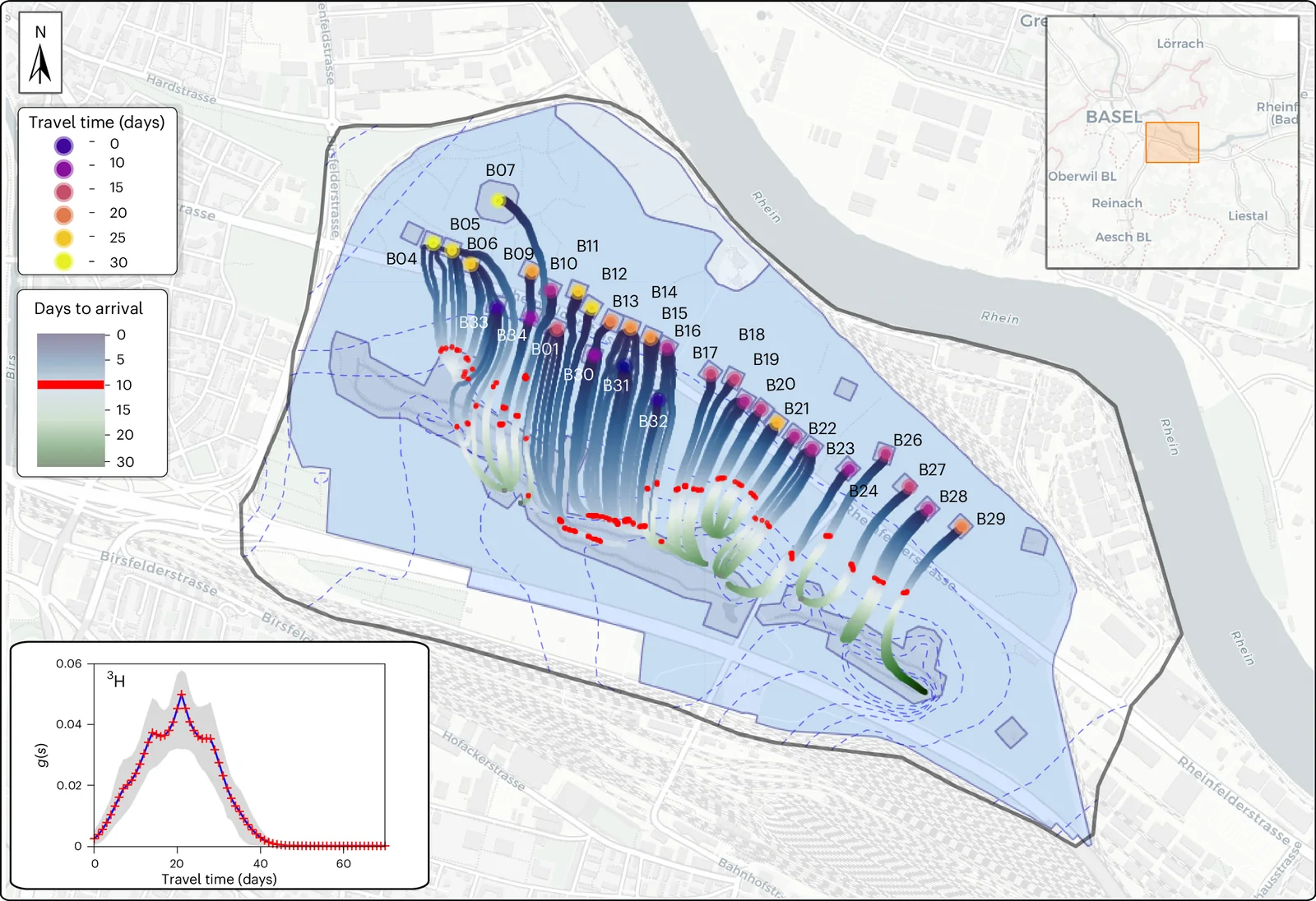
Travel time distributions derived from observed tracer levels across the Hardwald MAR scheme. The travel time per well computed using the groundwater transfer function (bottom left) and applied to the derived flow path from infiltration to each abstraction well. Travel times (as days to arrival of each peak) are represented as a shaded gradient along the flow path, with the 10-day point highlighted in red. Current groundwater protection areas (S1 and S2) are represented by shaded blue regions. Basemap tiles from CartoDB under a Creative Commons license CC BY-SA 2.0 with map data from OpenStreetMap under an Open Data Commons Open Database License (https://opendatacommons.org/licenses/odbl).

To further validate the deconvolution method and assess predictive accuracy, the ^3^H-derived travel time transfer function (Fig. 4) was mathematically integrated to predict the arrival time of infiltrated water to 30 abstraction wells within the Hardwald MAR system. Observed ^3^H abundances measured at these wells approximately 2 weeks after a distinct NPP release event in May 2023 (20–26 May 2023; Fig. 5) provided well-specific validation. The travel times of infiltrated water abstracted from wells located near the centre of the domain (for example, wells B31, B32 and B33), as predicted by deconvolution, are on the order of approximately 10 days (Fig. 5), which approaches the limits of Swiss wellhead protection zone area regulation^65^. Consequently, current abstraction protocols at these central locations may require revision to ensure longer residence times and safeguard water quality. Conversely, abstraction wells near the system perimeter with longer groundwater flow paths could be safely pumped at higher rates without compromising MAR system integrity, provided that the groundwater mound is maintained to avoid contamination by regional inflows.

The travel time distributions provide an operational basis for delineating wellhead protection areas and informing water safety management in MAR or natural groundwater systems along any large river with detectable ^3^H or d-excess variability. By resolving both rapid first arrivals (days) and longer peak and mean travel times (weeks), the approach supports risk-based wellfield management that maintains minimum residence times while preserving storage and recovery efficiency. As these tracers are intrinsic to the water molecule, the framework developed herein is safe and compatible with routine isotope monitoring, producing defensible travel time estimates that can be mapped onto capture zones and evaluated against existing regulatory thresholds such as the Swiss S2 criteria and analogous provisions under the EU Water Framework Directive. More broadly, the workflow addresses increasing national and transboundary requirements for quantitative evidence of groundwater travel times, vulnerability and protection zone effectiveness, particularly where recharge is engineered or induced. By translating high-frequency isotope monitoring into explicit travel time metrics that are directly comparable across sites, the method supports preventive management approaches embedded in water safety planning and contributes to broader objectives related to groundwater protection and sustainable drinking water supply. From a research perspective, it provides a transferable framework for harmonized time-series monitoring and travel time inference in river–groundwater systems, enabling systematic intercomparison and supporting the evaluation of groundwater flow and transport models under changing hydrological conditions.

## Conclusions

The travel time distributions derived from the deconvolution of the NPP ^3^H and the natural d-excess time series emerge as key tools in delineating wellhead protection areas and informing water safety management in MAR systems along the Rhine as well as along many of the major river systems worldwide (Fig. 1). The insights support a risk-based approach to MAR wellfield management, where abstraction rates are adjusted on the basis of estimated travel times to maintain both water quality and water preservation (for example, the Swiss wellhead protection area regulation or the EU Water Framework Directive). These results have important implications for the design and operation of MAR systems, and in fact, any groundwater system with observable ^3^H variations, offering a globally transferable, non-invasive, data-driven means to quantify subsurface travel times in MAR systems without artificial tracers. Through the integration of temporally highly resolved environmental isotope monitoring and non-parametric deconvolution, tracer-based transfer functions effectively reconstruct the travel times of infiltrate waters to the abstraction wells, aided by the fact that the tracers used are not solutes like other common tracers but part of the water molecule itself. As our capacity to accurately model ^3^H input continues to improve, this approach may also become viable in systems without direct NPP influence. The ability to quantify flow path variability and groundwater travel time distribution has direct implications for delineating wellhead protection zones, calibrating risk models and informing recharge management under climate and demand uncertainty. By enabling defensible estimates of groundwater travel times, this approach supports compliance with regulatory frameworks such as the EU Water Framework Directive and national groundwater protection policies. More broadly, it advances a transferable toolkit for optimizing MAR operations worldwide, particularly in data-limited or regulatory-sensitive environments.

## Methods

### Sample site, collection and analysis

The present study was carried out at the Hardwasser MAR scheme located along the banks of the Rhine River 5 km upstream of Basel in northwestern Switzerland. Hydrogeologically, the system consists of an alluvial aquifer composed of unconsolidated sediments, underlain by the highly heterogeneous regional Muschelchalk karst aquifer^66,67^. Such heterogeneity governs the spatial distribution of recharge rates and influences the transport of solutes and tracers. Previous studies have demonstrated how the interaction between the graben-controlled Muschelchalk with the overlying alluvium shapes the aquifer’s response to both natural and managed recharge inputs^47,48,68,69^. The MAR scheme is supplied by artificial infiltration through a network of ponds and canals and abstracted via 30 production wells that pump at relatively constant rates year-round, creating a transient steady state. Groundwater levels observed in a piezometer at the infiltration point fluctuate between 258.9 and 261.4 metres above sea level (MASL) with an annual average of 260.5 MASL. Similarly, groundwater levels in the production borehole fluctuate between 253.6 and 256.0 MASL with an annual mean of 255.17 MASL, resulting in an average hydraulic gradient of 0.012–0.018 over an average flow distance of 440 m between infiltration and abstraction points. According to literature, the average hydraulic conductivity of the quaternary unconsolidated sediments is 267 m per day, while that of the upper Muschelchalk aquifer it is 11 m per day^60^.

Infiltrate waters were collected using an ISCO autosampling unit set to collect 1 litre of water once a day at 9:00 every day from a flow-through collection tank in the water pretreatment facility of the Hardwasser MAR scheme (Fig. 2). Groundwater was collected in a similar fashion during the pumping hours of the production borehole at well-point B17 (Fig. 2). ISCO sample bottles were collected and restocked every 3 weeks. Both collection points are cool, dry rooms, retaining sample integrity during these periods. Collected samples were filtered (0.22 µm) into 300-ml polypropylene bottles and stored <4 °C before analysis (<3 months from collection date).

Stable isotopes of oxygen and hydrogen are generally referred to in delta notation (δ^18^O and δH^2^) in reference to Standard Mean Ocean Water (SMOW) using the appropriate accepted standards and steps to ensure accurate data acquisition^70^. The analysis of stable water isotopes (δ^18^O and δ^2^H) was conducted using cavity ring-down spectroscopy on a Picarro L-2140i at the University of Lausanne and at Eawag, Switzerland. Before measurement, all water samples were filtered (0.22 µm) and stored in air-tight glass vials to prevent evaporation and isotopic fractionation. The Picarro L-2140i operates by measuring the absorption of laser light within an optical cavity, enabling precise determination of isotopic ratios based on differential absorption spectra. Each sample was injected multiple times (ten replicates), with the first four injections discarded to eliminate memory effects. Calibration was performed using three in-house standards, normalized to the Vienna Standard Mean Ocean Water-Standard Light Antarctic Precipitation (VSMOW-SLAP) scale, ensuring comparability with international reference materials. Instrument precision was ±0.05‰ for δ^18^O and ±0.3‰ for δ^2^H, with accuracy verified through routine analysis of certified reference waters. Drift correction was applied using internal standards analysed periodically throughout the sample runs. To assess reproducibility, 10% of samples were analysed in duplicate, with deviations consistently within the instrument’s stated precision limits.

Similarly, careful procedures were applied for the collection, analysis and reporting of tritium (^3^H) activities. Tritium concentrations in water samples were determined by low-level liquid scintillation counting (LSC) at the University of Bern, Switzerland. Measurements were performed using a large-volume, ultralow-background liquid scintillation spectrometer (Aloka LSC-LBIII) equipped with active and passive background reduction. Additional background suppression is achieved by housing the instrument in the University of Bern’s underground laboratory, located approximately 46 m below the surface in sandstone bedrock, corresponding to ~75 m water-equivalent shielding. This reduces the cosmic-ray-induced muon flux by a factor of ~11 (ref. ^71^).

For each measurement, approximately 70 g of water was mixed with 60 g of Ultima Gold scintillator in 150-ml vials. After dark storage for about 1 week to allow stabilization, each vial was measured repeatedly (six to ten counting intervals of 100 min each), resulting in total counting times of roughly 1 week per sample. Beta decay events were recorded using a multichannel analyser over a linear energy range of 0–180 keV, with tritium evaluated within the characteristic energy window of its 18.6 keV beta spectrum. Instrument calibration was carried out using internal tritium standards covering the relevant activity range, and additional reference standards (0–100 TU) were regularly measured to monitor counting efficiency and background stability. This approach yields a detection limit of 2–4 TU without the need for electrolytic enrichment. Measurement uncertainty was derived from counting statistics and variability among repeated measurements, yielding typical uncertainties of approximately ±1.8 TU for the sample masses and counting times applied here. Measured count rates were corrected for radioactive decay between sampling and analysis, and background contributions were quantified using blank samples^72^. The analytical performance of the Aloka LSC LBIII system and the applied calibration and correction procedures have been independently validated in interlaboratory proficiency tests and documented in previous methodological studies^48,52,73^.

### Peak correlation and time-series deconvolution

To quantify travel times and characterize transport behaviour within the MAR system, we implemented a statistical framework consisting of (1) peak-matching and cross-correlation analysis, (2) non-parametric deconvolution of tracer time series to derive system transfer functions, and (3) spatial application of these transfer functions to ^3^H records across 30 observation wells. All statistical analyses were conducted in R using adapted custom routines based on refs. ^45,46^. Initial travel time estimates were made using a threshold-based peak detection algorithm^61^, followed by lagged Pearson correlation to identify the delay between infiltrate and abstraction signals^74^. While this approach identifies temporally aligned signal responses from infiltrated to abstracted time series, it provides only an approximate measure of travel time and does not capture the shape or uncertainty of the full tracer distribution. To more robustly characterize flow, travel times, mixing and dispersion, we applied non-parametric deconvolution to 1-year, high-resolution infiltration and abstraction tracer time series from a single well with full coverage for two suitable tracers (^3^H, d-excess). In this framework, the measured abstraction signal *C*_abs_(*t*) is assumed to be the convolution of the infiltration signal *C*_inf_(*t*) with a transfer function *g*(*s*):

$${C}_{\mathrm{abs}}\left(t\right)={\int }_{0}^{t}g\left(s\right){C}_{\inf }\left(t-s\right){\rm{d}}s,$$

where *g*(*s*) is the unitless tracer-based transfer function, which in the case of an MAR system as described herein translates into the probability density function (PDF) of groundwater travel times from the infiltration point to the abstraction well. It describes the system’s response to an instantaneous unit input (that is, a delta function). As such, the transfer function is not equivalent to the breakthrough curve itself but can reproduce it when convolved with a continuous tracer signal. Importantly, deconvolution does not reconstruct the breakthrough curve directly; rather, it isolates the underlying transport structure by evaluating how any point in time in the infiltration record is translated to the abstraction point via the PDF of travel times. The tracer-based transfer function is determined by minimizing the sum of squared residuals between the modelled convolution *C*_inf_ and the observed data *C*_abs_. To stabilize the inverse problem, a Tikhonov-regularized least-squares minimization was used (as previously described by ref. ^46^):

$$\frac{\min }{g(s)}{{||}{C}_{\inf }\times g(s)-{C}_{\mathrm{abs}}{||}}^{2}+\lambda {\mathrm{||}\nabla g(s)\mathrm{||}}^{2}$$

The first term (|*C*_inf_ × *g*(*s*) − *C*_abs_|^2^), quantifies the discrepancy between modelled (*C*_inf _× *g*(*s*)) and observed (*C*_abs_) breakthrough curves, aiming to ensure fidelity to the measured data. The second term, (*λ*||∇*g*(*s*)||^2^), enforces a smoothness constraint on the transfer function by penalizing large spatial gradients, thus preventing overfitting. The regularization parameter *λ* was optimized individually for each tracer via cross-validation. Deconvolution was performed independently for each tracer and repeated across 1,000 Monte Carlo realizations incorporating input noise to generate an ensemble of plausible transfer functions. From this ensemble, we derived mean estimates and 1 − *σ* confidence intervals. The best non-negative estimate, corresponding to the optimal unconstrained solution, is indicated along the generated transfer function. Transport metrics (Supplementary Table 2) were extracted from each transfer function, providing a basis to evaluate tracer-specific sensitivity to dispersion and attenuation. Among all tracers, the ^3^H-derived transfer function showed the highest fidelity during convolution-based reconstruction (Supplementary Fig. 2), probably due to its pulse-like character and low background variability. For this reason, the ^3^H-derived transfer function was selected for spatial application.

To assess the spatial distribution of groundwater travel times within the Hardwald MAR system, we applied the transfer function derived from the ^3^H tracer at the reference well-point B17 to a network of 30 spatial observation wells. Among all tested tracers, ^3^H produced the most consistent reconstruction results and was therefore selected to represent the system-wide travel time response. Using this validated transfer function, we conducted forward modelling (reconvolution) to simulate expected breakthrough curves across the spatial observation network. This involved convolving the known ^3^H input signal from infiltrate water with the transfer function to generate a predicted time series at each well location. Observed ^3^H concentrations from a coordinated spatial sampling campaign (July 2023) were then matched to the simulated output signal by identifying the best-fit arrival time (*t*_arrival_), defined as

$${t}_{\mathrm{arrival}}={\arg }_{t}^{\min }|{C}_{3{\rm{H}},\mathrm{sim}}\left(t\right)-{C}_{3{\rm{H}},\mathrm{obs}}|.$$

This estimated arrival time represents the tracer’s best-matching appearance at each spatial point, assuming consistent transport behaviour governed by the reference transfer function. To translate these arrival times into spatially continuous capture zones, we integrated the transfer function with backward particle tracks from an existing steady-state groundwater flow model of the study site^75^. Flow paths originating at the abstraction wells were extracted under average recharge and pumping conditions. Each path was uniformly resampled at 2-m intervals, producing discrete spatial nodes from the infiltration area to the abstraction well. For each flowline, the total length was divided by the inferred ^3^H-based travel time to calculate an average advective velocity. This velocity was used to assign cumulative travel times along each resampled flowline. The result is a spatially continuous field of travel times that honours both the deconvolution-derived timing and the modelled subsurface flow direction and path. From this distribution, isochrones representing 10-day capture zones were delineated, enabling operational assessment and visualization of MAR performance. This hybrid geospatial–statistical workflow enables a physically coherent integration of signal-based travel time inference with hydrodynamic transport structure, supporting both management and regulatory applications.

### Limitations

While the deconvolution technique provides valuable insights into travel time distributions, several limitations and uncertainties must be acknowledged. First, the method assumes a linear and time-invariant system, which may not fully capture the dynamic variability of infiltration and abstraction rates observed in operational MAR schemes. Furthermore, the accuracy of the derived travel time depends on the temporal resolution and quality of the tracer time series; this is evident when daily resolution observations are averaged over longer periods and the deconvolution deteriorates to the point that a PDF cannot be computed (>9 days) (Supplementary Fig. 1). This outcome suggests that, under the conditions of this study, compound sampling over multiday intervals does not improve the estimation of groundwater travel time distributions using stable isotopes, and the most efficient sampling resolution would be between 5 and 9 days. The interpretation of groundwater travel times using tracer time-series deconvolution is also sensitive to assumptions about mixing behaviour, although the deconvolution technique itself does not rely on assumptions about transport behaviour and instead translates transport into a PDF of travel times. It is important note that the deconvolution computation is ‘non-parametric’ and does not incorporate any physical boundaries of the actual system. Furthermore, while ^3^H is treated here as a quasi-conservative tracer, its decay and potential retardation under certain conditions should be considered when extrapolating travel times over longer durations. These uncertainties highlight the importance of integrating deconvolution results with other hydrogeological observations, including pumping tests and numerical flow models, to build a more comprehensive understanding of system behaviour.

### Reporting summary

Further information on research design is available in the Nature Portfolio Reporting Summary linked to this article.

## Supplementary information

Supplementary Information Supplementary Discussion, Tables 1–3 and Figs. 1 and 2.

Reporting Summary

Peer Review File

## Data Availability

Records of tritium in rivers, and the exact locations of the stations considered in this Article, can be accessed through the International Atomic Energy Agency (IAEA) Global Network of Isotopes in Rivers (GNIR) database at https://nucleus.iaea.org/wiser. The measured isotope time series presented in this publication can be accessed via CUAHSI’s online collaboration platform, HydroShare, at http://www.hydroshare.org/resource/e3b0781994ed447cad10364e9c880652.
