# Supplementary material for: Anthropogenic tritium as a continental-scale tracer in river-derived recharge
Source: Nat Water. 2026 Mar 23;4(4):444–54. doi: 10.1038/s44221-026-00616-x (PMC13652735; doi:10.1038/s44221-026-00616-x)
Supplement: Supplementary file 1 [file 44221_2026_616_MOESM1_ESM.pdf]

nature water

Supplementary information https://doi.org/10.1038/s44221-026-00616-x

Anthropogenic tritium as a continental-scale tracer in river-derived recharge

In the format provided by the authors and unedited

Supplementary Material

Global Distribution and Suitability of River Basins for Nuclear Power Derived Tracer Analyses

Our analysis of the global distribution of operating and planned nuclear power plants (NPPs) alongside the distribution of managed aquifer recharge (MAR) schemes affirms the suitability of using tritium released from NPPs as environmental tracer for the study of MAR performance along many major river basins on Earth (Fig. 1). Our assessment focused on major river basins where (i) MAR systems and NPPs coexist, (ii) the hydrological connectivity supports downstream transport of released tritium, and (iii) MAR sites are positioned to intercept these signals for potential tracer studies.

The Danube Basin emerges as the highest-ranked region for applying NPP-derived tracers to managed aquifer recharge (MAR) systems. Numerous operating NPPs (e.g., Temelín, Paks, Kozloduy, and Cernavodă) are situated along the mainstem or major tributaries. Key MAR schemes, especially induced bank filtration systems near Vienna, Budapest, and downstream sites in Romania, are located along the downstream path of these reactors. Historic studies have documented ^3H signals from upstream nuclear facilities in this basin, and the presence of low-background groundwater provides a favourable contrast for tracer resolution [1,2]. The Danube's well-monitored hydrology and MAR infrastructure make it an optimal testbed for further NPP tracer applications. In Western Europe, the Rhine and Elbe basins also present strong candidates. Operating NPPs in France (e.g., Fessenheim), Switzerland (e.g., Beznau), and Germany (e.g., Grohnde) discharge into upstream reaches of these basins. These rivers host extensive MAR infrastructure, including surface spreading systems and induced bank filtration (e.g., Basel, Düsseldorf, Hamburg). Past environmental ^3H monitoring confirms detectable downstream signal propagation [3–8]. Compared to the Danube, however, regulatory constraints and river engineering (e.g., weirs, locks) may modify tracer continuity. Nonetheless, the overall density and monitoring maturity of these systems rank them highly in terms of NPP tracer-MAR synergy.

The Mississippi Basin in the United States shows moderate potential. While NPP density is high in the Midwest and southeastern portions (e.g., Clinton, Grand Gulf, and Watts Bar stations), most MAR schemes, especially surface spreading and recharge wells, are located in the arid southwest, far from major reactors. Exceptions include induced bank filtration sites in Illinois and Texas, where some downstream alignment exists. However, vast distances and dilution from the expansive drainage network limit the applicability of NPP-derived signals in most western MAR settings. Thus, only select sites offer promise, and tracer resolution would benefit from coupling with other isotopic or hydrochemical markers.

In India's Ganges Basin, NPPs like Narora and Kudankulam are often situated upstream or near densely populated zones where MAR has been implemented to combat water scarcity. Induced bank filtration schemes along the Ganges and Yamuna rivers lie directly downstream of several NPPs. While tritium release data are limited, historic radiological surveys suggest measurable background shifts that could serve as diffuse tracers [9]. The large water turnover, episodic recharge events, and unregulated urban flows, however, introduce substantial variability, which may obscure direct signal propagation. Nevertheless, the spatial coupling of NPPs and MAR infrastructure suggests tracer applicability, particularly when paired with Bayesian reconstruction methods. The Amu Darya–Syr Darya basin in Central Asia, while less densely populated, shows increasing MAR development driven by climate adaptation

programs [10,11]. NPPs in Kazakhstan and Uzbekistan are planned or under construction along major rivers feeding this basin. The opportunity lies in real-time tracer integration from the outset of NPP operation and MAR development. As such, this region represents a high-future-potential zone where proactive tracer calibration and hydrological monitoring could yield significant insights for tracer-based MAR tracking.

In Eastern Asia, a high density of nuclear power plants is only found along coastal areas. Yet in China, ^{3}H releases from the Qinshan and Daya Bay nuclear complexes coincide with estuarine systems that are influenced by oceanic release waters. However, tracer applicability may be constrained by already elevated ^{3}H backgrounds in the region [12,13] and complex hydro-engineering infrastructure that mediates river-aquifer interactions. Still, the presence of large, long-established NPPs with known release histories supports the viability of NPP-derived tracers in these regions, particularly during peak discharge periods. Regions such as South Korea and Japan also merit attention. The Han River Basin, with the Kori and Wolsong NPPs, discharges into estuarine environments near MAR sites that employ different MAR methods [14,15]. Here, the high seasonal variability may obscure long-term tracer retention but presents opportunities for assessing shorter-term travel times. Likewise, in Japan's Tone and Shinano River Basins, upstream nuclear facilities coexist with MAR schemes that support urban water security. Although public reporting of tritium release is more restricted, peer-reviewed literature documents sufficient release levels for tracer utility [16].

Some emerging regions should also be of interest, including East Africa, particularly along the Nile and Tana River Basins, where MAR schemes are expanding in tandem with nuclear infrastructure planning (e.g., Kenya's Kilifi NPP project). While there currently are no existing NPPs, the strong planning signals and clear hydrological dependencies suggest that future tracer experiments could be designed with ideal configurations for downstream tracer studies. Until reactors come online, simulations using surrogate tracers and modelling can guide the design of MAR-suited isotope monitoring frameworks. Similarly, in South America, the La Plata Basin (especially Paraná and Uruguay Rivers) hosts both operational NPPs (e.g., Atucha, Embalse in Argentina) and MAR systems being piloted for drought mitigation. The basin's confined flow paths and moderate recharge variability make it a promising candidate. However, the relatively low density of MAR installations limits immediate scalability. If national programs expand water banking, especially in Uruguay and southern Brazil, tracer-based assessments could become central to system performance evaluations.

Together, these observations reveal that successful application of NPP-derived tracers to MAR hinges on four key factors: (1) upstream positioning of NPPs relative to MAR systems, (2) sufficient proximity to limit tracer dilution, (3) presence of hydrological continuity between release and abstraction points, and (4) stable background isotope levels for signal contrast. Among these, the Danube and Rhine Basins currently offer the most reliable real-world conditions, while systems in East Asia, South Asia, and South America present emerging opportunities. Future efforts should prioritize long-term tracer monitoring in these basins to assess signal persistence and hydrogeochemical behaviour under operational MAR conditions.

Effect of Temporal Aggregation on Transfer Function Estimation for Stable Isotopes and ^{3}H

To evaluate the sensitivity of non-parametric deconvolution to the temporal resolution of sampling, we conducted a binning experiment using stable water isotopes and ^{3}H. This analysis aimed to determine the optimal sampling frequency or sample aggregation interval of easily measured tracers ($\delta^{18}O$, $\delta^{2}H$) and more demanding tracers (^{3}H) by taking the observed tracer

time series and binning them into longer compound intervals (from 1 to 3, 5, 7, 9 and 11 days for $\delta^{18}O$, $\delta^{2}H$; from 1 to 2 and 3 weeks for ^{3}H). Each binned dataset was subjected to the same deconvolution algorithm used in the primary analysis, and resulting transfer functions were compared across binning scales. Besides aiming to optimize sampling and analysis frequency, we furthermore hypothesized that short-term signal variability in $\delta^{18}O$ and $\delta^{2}H$, potentially related to weather events or operational, could be smoothed out such that $\delta^{18}O$ and $\delta^{2}H$ could be used to achieve similar results as deuterium excess (d-excess) and ^{3}H. Aggregating $\delta^{18}O$ and $\delta^{2}H$ data to a sufficiently long interval was thus suspected to reduce noise and improve the physical interpretability of deconvolution based transfer functions of $\delta^{18}O$ and $\delta^{2}H$. However, as shown in Supplementary Fig. S1, increasing the binning interval did not significantly enhance the clarity or coherence of the transfer functions derived from $\delta^{18}O$ and $\delta^{2}H$.

The forms of the transfer functions for $\delta^{18}O$ and $\delta^{2}H$ remained largely consistent across binning scenarios, and estimated travel time metrics (t_peak, t_c) showed limited convergence. The expected gains in smoothness or peak resolution were not evident. Conversely, for d-excess, some minor reduction in high-frequency variation was observed, yet peak time and center of gravity estimates still varied with binning. Notably, at 9-day and 11-day intervals, the Laplace inversion step became numerically unstable and could not produce a reliable transfer function anymore, likely due to loss of temporal detail necessary to resolve transport-related signal progression.

For ^{3}H, as the observations were collected as composite weekly aggregate samples, a multiple week binning was attempted but no non-zero function could be derived from multi weeks bins, suggesting that even a two-week aggregate would not suffice in delineating representative deconvolution results. The outcome thus reveals that, at least under the conditions of this study, the optimal sampling aggregation interval for d-excess and ^{3}H is between 5 and 9 days, thus rendering daily or sub-weekly sampling of stable water isotopes and ^{3}H unnecessary for a robust analysis of travel times in alluvial MAR schemes.

Time Series Reconstruction Validation

To validate the transfer functions derived from the tracer time series (Fig. 3), we applied a convolution-based reconstruction procedure using the observed infiltration signals and the non-parametrically estimated transfer functions g(s) for d-excess and ^{3}H. The goal of this step was to evaluate whether the inferred travel time distributions captured the system's transport dynamics with sufficient accuracy to reproduce observed abstraction point. When convolved with any input concentration time series, the transfer function yields the expected tracer concentration at the abstraction point, assuming linear and time-invariant conditions. While estimated from full input-output time series, the function characterizes the aquifer’s internal mixing and transport by describing how each moment in the input contributes probabilistically to the output signal. Importantly, the transfer function is not equivalent to the breakthrough curve itself, except in the special case of a delta-like (instantaneous) input, but it enables reproduction of breakthrough curves when applied to real-world tracer behaviour. To test the fidelity of this transfer-function-based reconstruction, the tracers’ infiltration time series were convolved with their corresponding transfer function. Prior to convolution, a synthetic 60-day “preconditioning” period was appended to the input signal using default background concentrations, ensuring physically plausible boundary conditions and preventing artificial signal truncation. The resulting outputs, i.e., the reconstructed abstraction time series, were then compared directly to the observed signals for the two tracers. To capture uncertainty similar to the derivation of the original transfer function, Monte Carlo realizations of the deconvolution were propagated through convolution, yielding 16th–84th percentile bounds on

the reconstructed output. This confidence envelope reflects variability in the estimated travel time distribution itself, rather than analytical measurement error.

Results demonstrated varying levels of agreement between reconstructed and observed signals (Fig. S2a), which were assessed visually and statistically. Linear regression between predicted and observed values (Fig. S2b) yielded coefficients of determination (R^2) of 0.606 for ^3H, and 0.128 for d-excess. The transfer function derived from ^3H showed the strongest performance, accurately reproducing both the amplitude and timing of observed signals. The differences in reconstruction performance are attributed not to intrinsic transport behaviour of the tracers, which undergo the same physical processes in the aquifer, but rather to the structure of the input signals. The ^3H record displayed sharp pulses and low background noise, ideal conditions for extracting coherent transfer functions. In contrast, the d-excess record exhibited smoother long term (seasonal) gradients, complicating the interpretation as transport indicator. These findings support the suitability of non-parametric deconvolution for characterizing travel time distributions under MAR conditions. While stable isotopes provide valuable insight into source mixing and long-term trends, at least when looking at the derived d-excess metric, the ^3H tracer offers higher resolution for reconstructing transport timing, making it especially well-suited for applications such as wellhead protection, system diagnostics, and performance monitoring under dynamic flow conditions.

Summary table of NPPs and MAR sites in large river basins

Table S1. Summary of basin rankings for implementation of NPP released tracer studies in MAR/River fed groundwater studies.

RANK	RIVER BASIN	KEY NPPS	MAR METHODS	TRACER APPLICABILITY COMMENTS
1	Danube (Europe)	Mochovce, Bohunice, Paks	induced bank filtration, well injection	Upstream NPPs with known ^{3}H discharge; bank filtration sites downstream
2	Rhine (Europe)	Beznau, Grösgen, Fessenheim	induced bank filtration, surface spreading	Strong historical ^{3}H signal; validated at MAR sites like Hardwald
3	Han (South Korea)	Kori, Wolsong	well injection, subsurface dams	Seasonal flow challenges but proximity favors short-term tracer use
4	Mississippi (USA)	Grand Gulf, River Bend	spreading methods, well injection	High flow dilution; NPP upstream; suitable under steady flow
5	Yellow River (China)	Hongyanhe, Tianwan	In-channel modifications, surface spreading	Similar to Yangtze but with more hydrological complexity
6	Ganges (India)	Narora, Kakrapar	In-channel modifications, induced bank filtration	Limited MAR documentation; ^{3}H detected downstream of NPPs
7	Great Lakes, Saint Lawrence (Canada/USA)	Bruce, Pickering, Darlington	surface spreading, well injection	High ^{3}H release from CANDU reactors; MAR pilots emerging
8	Tone (Japan)	Kashiwazaki-Kariwa	well injection, indirect recharge	Urban basin; less reported ¬≥H data; potential in short-term studies
9	Sacramento Joaquin (USA)	Diablo Canyon (indirect influence via flow paths)	Surface spreading, In-channel modifications	Less consistent NPP influence, but tracer potential during discharge events
10	Yangtze (China)	Qinshan, Taishan, Daya Bay	Surface spreading, induced bank filtration	High upstream ^{3}H input; dense MAR zones; strong hydrological link

Summary table of transfer function metrics

Table S2. Summar of transfer function metrics. Abbreviations: T = truncation time; ng = number of estimated values; m0g = zeroth moment (recovery rate); t0g = time of first breakthrough; tcg = center of gravity; tpeak = peak arrival time; sigma_g = standard deviation of determined travel-time distribution (measure of spread); Err = uncertainty of computation.

tracer	T (days)	ng	m0g (%)	t0g (days)	tcg (days)	tpeak (days)	sigma g (days)	Err (days)
d-excess	58.76	99	0.99	0	26.87	21.39	11.88	5.92
^{3}H	42.44	71	0.99	1	20.19	21.00	8.5	4.79

Summary table of spatially derived travel times

Table S3. Summary statistics of spatial integration results for all 30 wells within the Hardwald MAR scheme, including the date the sample was collected, the average distance of all flow paths from the infiltration point to the observation abstraction point (well), the measured ^{3}H value, the computed peak travel time (PTT) and associated error (PTT Err), the peak groundwater flow velocity (PV), the mean travel time of all flow paths (MTT) and associated error (MTT Err) and mean travel velocity (MV).

EID	*Sample date* (DD/MM/YY)	*Distance* (m)	*^{3}H* (TU)	*PTT* (day)	*PTT Err* (day)	*PV* (m/d)	*MTT* (day)	*MTT Err* (day)	*MV* (m/d)
B01	12/06/2023	690.60	28.92	21.0	3.5	32.89	39.0	6.5	17.71
B04	13/06/2023	1064.60	22.48	30.0	3.2	35.49	42.0	4.5	25.35
B05	13/06/2023	1018.90	23.55	29.0	3.9	35.13	41.0	5.5	24.85
B06	13/06/2023	959.40	24.22	28.0	4.1	34.27	41.0	6.0	23.40
B07	13/06/2023	1048.10	22.87	30.0	3.2	34.94	42.0	4.5	24.95
B09	13/06/2023	849.00	26.15	26.0	5.1	32.65	41.0	8.0	20.71

B10	06/06/2023	780.50	26.76	18.0	4.1	43.36	33.0	7.5	23.65
B11	13/06/2023	740.30	24.36	28.0	4.1	26.44	41.0	6.0	18.06
B12	13/06/2023	688.90	23.88	29.0	3.9	23.76	41.0	5.5	16.80
B13	13/06/2023	634.40	27.39	24.0	4.2	26.43	40.0	7.0	15.86
B14	13/06/2023	599.30	26.71	25.0	4.7	23.97	40.0	7.5	14.98
B15	13/06/2023	558.70	27.26	25.0	4.7	22.35	40.0	7.5	13.97
B16	06/06/2023	521.60	27.20	18.0	4.1	28.98	33.0	7.5	15.81
B17	06/06/2023	440.40	25.37	20.0	4.1	22.02	34.0	7.0	12.95
B18	06/06/2023	429.90	25.61	20.0	4.1	21.50	34.0	7.0	12.65
B19	06/06/2023	376.80	27.16	18.0	4.1	20.93	33.0	7.5	11.42
B20	06/06/2023	367.10	25.73	19.0	4.5	19.32	34.0	8.0	10.80
B21	12/06/2023	350.00	24.01	27.0	4.1	12.96	40.0	6.0	8.75
B22	06/06/2023	344.60	26.71	18.0	4.1	19.14	33.0	7.5	10.44
B23	06/06/2023	351.10	27.26	18.0	4.1	19.50	33.0	7.5	10.64
B24	06/06/2023	396.60	27.64	17.0	3.6	23.33	33.0	7.0	12.02
B26	06/06/2023	497.40	25.76	19.0	4.5	26.18	34.0	8.0	14.63
B27	06/06/2023	525.20	25.56	20.0	4.1	26.26	34.0	7.0	15.45
B28	05/06/2023	556.80	26.42	18.0	4.4	30.93	33.0	8.0	16.87
B29	12/06/2023	639.00	27.30	24.0	4.6	26.62	39.0	7.5	16.38
B30	12/06/2023	579.50	33.25	16.0	3.2	36.22			
B31	06/06/2023	515.70	33.13	10.0	5.1	51.57			
B32	06/06/2023	399.90	32.31	11.0	4.1	36.35			
B33	06/06/2023	831.30	31.23	12.0	4.1	69.28			
B34	06/06/2023	753.30	28.15	16.0	3.4	47.08	33.0	7.0	22.83
C18	07/06/2023	1049.90	10.01	35.0	3.4	30.00	41.0	4.0	25.61
C206	07/06/2023	1164.00	9.32	35.0	3.4	33.26	41.0	4.0	28.39
C222	07/06/2023	506.70	17.00	29.0	3.1	17.47	38.0	4.0	13.33
C227	07/06/2023	926.00	25.47	21.0	4.2	44.10	35.0	7.0	26.46
C228	07/06/2023	471.10	13.08	32.0	3.3	14.72	39.0	4.0	12.08
C229	07/06/2023	751.00	10.93	34.0	2.9	22.09	41.0	3.5	18.32
C230	07/06/2023	894.20	9.07	36.0	3.0	24.84	42.0	3.5	21.29
C43	07/06/2023	271.60	19.82	26.0	2.9	10.45	36.0	4.0	7.55
C80	07/06/2023	816.80	23.16	23.0	3.6	35.51	35.0	5.5	23.34
C81	07/06/2023	601.60	23.64	23.0	3.6	26.16	35.0	5.5	17.19
C83	07/06/2023	648.40	19.29	27.0	2.9	24.01	37.0	4.0	17.52

Supplementary Figures

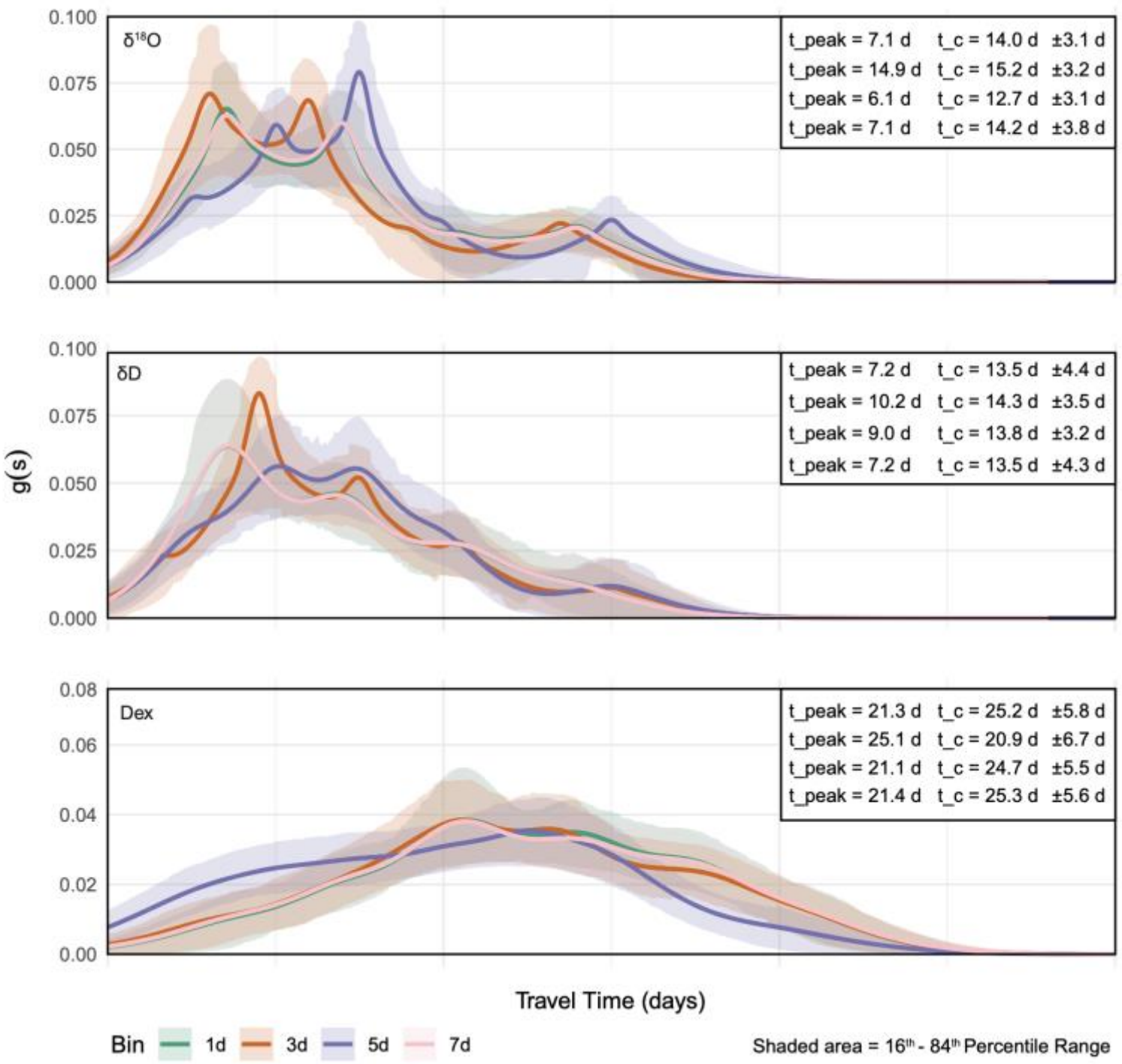

Figure S1. Influence of temporal aggregation on deconvolution-derived transfer functions for δ¹⁸O, δ²H, and d-excess (n=358). The Fig. shows the non-parametric transfer functions g(s) derived from infiltrate and abstraction signals after binning the original daily observations into 1-, 3-, 5-, and 7-day intervals. Transfer function curves (solid lines) are shown with their associated uncertainty envelopes (shaded areas), and tabulated metrics report peak travel time (t_peak), center of gravity (t_c), and associated uncertainty for each binning scenario. The Laplace inversion becomes unstable beyond 7-day binning (not shown).

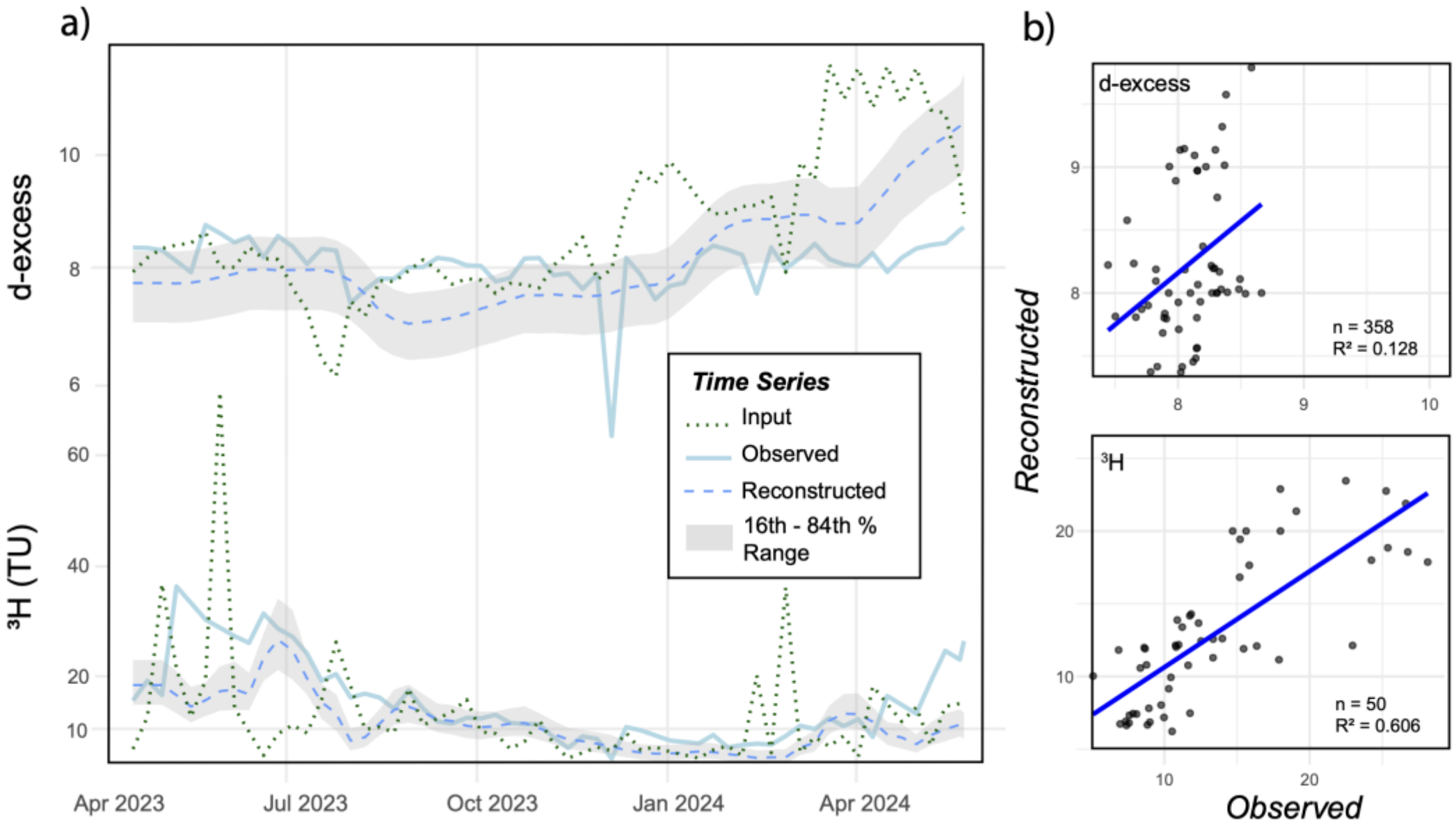

Figure S2. Validation of groundwater transfer function reconstructions for four environmental tracers. a) panels show time series of tracer concentrations from the infiltration source (dotted), observed groundwater (solid), and transfer function-based reconstructions (dashed), with uncertainty envelopes derived from the 16th–84th percentile range of the transfer function ensemble. Transfer functions were applied via convolution using tracer-specific input signals under the assumption of linear and time-invariant system behaviour. b) panels show reconstructed versus observed concentrations for d-excess and ³H, with 1:1 aspect ratio and fitted linear regression lines (blue).

References (Supplementary Material)

1. Grahek, Ž. *et al.* Measurement of tritium in the Sava and Danube Rivers. *J Environ Radioact* **162–163**, 56–67 (2016).

2. Janković, M. *et al.* Natural or Artificial Tritium in Rivers – the Assessment using Symmetrical Index. *Nuclear Engineering and Design* **427**, (2024).

3. Barešić, J., Parlov, J., Kovač, Z. & Sironić, A. Use of nuclear power plant released tritium as a groundwater tracer. *Rudarsko Geolosko Naftni Zbornik* **35**, 25–34 (2020).

4. Desyatov, D. D., Ekidin, A. A. & Vlasov, D. A. Global release of tritium into the environment: NPP reactor type contribution. *Atomic Energy* **137**, 198–205 (2025).

5. Vlasov, D. A., Desyatov, D. D. & Ekidin, A. A. Forecast of tritium release into the environment during the normal operation of nuclear power plants. *Atomic Energy* (2025) doi:10.1007/s10512-025-01241-9.

6. Schubert, M., Müller, C., Knoeller, K. & Juranová, E. Anthropogenic tritium as indicator for groundwater inflow into major rivers – Potentials and challenges of a tracer application. *J Environ Radioact* **288**, (2025).

7. Zill, J. *et al.* A way to determine groundwater contributions to large river systems: The Elbe River during drought conditions. *J Hydrol Reg Stud* **50**, (2023).

8. Desens, A., Houben, G., Sültenfuß, J., Post, V. & Massmann, G. Distribution of tritium-helium groundwater ages in a large Cenozoic sedimentary basin (North German Plain). *Hydrogeol J* **31**, 621–640 (2023).

9. Datta, P. S. *Isotope Fingerprinting of the Ganges Basin Groundwater to Protect It from Vulnerability to Depletion and Pollution*. https://www.researchgate.net/publication/301778384 (2009).

10. Wendt, D. E., van Loon, A. F., Scanlon, B. R. & Hannah, D. M. Managed aquifer recharge as a drought mitigation strategy in heavily-stressed aquifers. *Environmental Research Letters* **16**, (2021).

11. Zhao, M., Boll, J., Adam, J. C. & King, A. B. Can managed aquifer recharge overcome multiple droughts? *Water (Switzerland)* **13**, (2021).

12. Liang, J. *et al.* Distributions of tritium with different chemical form in the soil around Qinshan Nuclear Power Plant. *J Radioanal Nucl Chem* **328**, 1153–1159 (2021).

13. Nie, B. *et al.* Numerical study on tritium dispersion in coastal waters: The case of Hangzhou Bay, China. *J Hydrol (Amst)* **590**, (2020).

14. Cao, H. *et al.* Managed aquifer recharge and exploitation impacts on dynamics of groundwater level and quality in northern China karst area: Quantitative research by multi-methods. Preprint at https://doi.org/10.5194/egusphere-2025-281 (2025).

15. Lee, K. S. & Kim, Y. Determining the seasonality of groundwater recharge using water isotopes: A case study from the upper North Han River basin, Korea. *Environmental Geology* **52**, 853–859 (2007).

16. International Atomic Energy Agency. *IAEA Comprehensive Report on the Safety Review of the ALPS-TREATED Water at the Fukushima Daiichi Nuclear Power Station*. (2023).
