# Supplementary material for: Anthropogenic tritium as a continental-scale tracer in river-derived recharge
Source: Nat Water. 2026 Mar 23;4(4):444–54. doi: 10.1038/s44221-026-00616-x (PMC13652735; doi:10.1038/s44221-026-00616-x)
Supplement: Supplementary file 3 [file 44221_2026_616_MOESM3_ESM.pdf]

nature portfolio

Peer Review File

Anthropogenic Tritium as a Continental Scale Tracer in River Derived Recharge

Corresponding Author: Dr Jared van Rooyen

Version 0:

Reviewer comments:

Reviewer #1

(Comments for the Author)
Summary of the key results

This is a very interesting and valuable manuscript, as it demonstrates the practical applicability of tritium (^3H) released from nuclear power plants as a natural environmental tracer in Managed Aquifer Recharge (MAR) systems. The authors combine high-frequency sampling of $\delta^{18}O$, δ^2H, deuterium excess (Dex), and ^3H with non-parametric time-series deconvolution to reconstruct groundwater travel times in a large MAR system along the Rhine River. The results show that tritium released from NPPs provides a clear, quasi-conservative tracer signal, while Dex reflects seasonal meltwater dynamics and also functions as an effective tracer. The derived travel-time metrics support improved delineation of wellhead protection zones and optimization of MAR operational strategies.

Originality and significance

The work is significant and timely. Although the use of environmental tracers in MAR studies is well established, the explicit demonstration that routine tritium releases from nuclear power plants can be used as operational tracers is novel and highly relevant.

Data & methodology

The dataset is extensive, high-resolution, and carefully collected. The methodology—particularly the use of non-parametric deconvolution—is appropriate and clearly described. The high temporal resolution of the stable isotopes and ^3H is a major strength. The figures are convincing:

Figure 1: There is a data gap for tritium at Wesel between 2017 and 2020. WISER refers to the publication by Schmidt et al., which includes at least the data for 2018. I recommend incorporating these data and citing the publication accordingly.

Line 330 ff: The ability to measure tritium directly at such low concentrations is remarkable. The manuscript would benefit from a more detailed description of the analytical method and additional references, especially because the Aloka LSC_LBIII instrument may not be widely known within the tritium/LSC community.

Appropriate use of statistics and treatment of uncertainties

The statistical treatment appears appropriate, particularly the use of Monte Carlo realizations to quantify uncertainty in the transfer functions. The discussion of travel-time variability and the limitations associated with binning is transparent and well reasoned.

Conclusions: robustness, validity, reliability

The conclusions are well supported by the presented results. The agreement between the transfer functions derived from Dex and ^3H increases confidence in the robustness of the approach.

Suggested improvements

To further strengthen the manuscript, the following additions could be considered:

Clarification of the U-shaped tritium pattern in Figure 3 (for the abstracted water). Are older time series from the observation well available to help interpret this feature?

A more explicit discussion of how MAR system geometry (e.g., hydraulic gradient, variability in infiltration rates) may influence the deconvolution results.

References

The reference list is extensive and appropriate. However:

Citation 56 contains a formatting error and should be corrected.

Clarity and context

The manuscript is generally well written, and the abstract is clear and informative. The introduction presents the problem and novelty of the study in a coherent manner. The results section is logically structured and supported by well-designed figures. The conclusions are clear and consistent with the data.

Reviewer #2

(Comments for the Author)
The manuscript presents an interesting example of utilizing 3H in addition to water stable isotopes to infer groundwater travel times in a managed aquifer recharge system associated with the presence of effluents of nuclear power plants (NPP). The authors successfully managed to demonstrate the usability of 3H as travel time tracer where increases of 3H are present due to water discharges related to NPP, replicated also by the d-excess due to the seasonal meltwater signal.

I don't have any significant comments, apart for one concern on the relatively short observation period of 1 year for the identification of seasonal trends (e.g., in terms of d-excess), exemplified by the weak correlation in the validation test, and some minor technical remarks indicated in the annotated version attached.

I think this case study is a nice contribution and recommend acceptance after applying the minor corrections indicated.

Version 1:

Reviewer comments:

Reviewer #1

(Comments for the Author)
All comments and suggestions have been considered into the revised manuscript.

Reviewer #2

(Comments for the Author)
I'm satisfied with the modifications and responses provided by the authors.
I recommend publication.

Open Access This Peer Review File is licensed under a Creative Commons Attribution 4.0 International License, which permits use, sharing, adaptation, distribution and reproduction in any medium or format, as long as you give appropriate credit to the original author(s) and the source, provide a link to the Creative Commons license, and indicate if changes were made.
In cases where reviewers are anonymous, credit should be given to 'Anonymous Referee' and the source.
The images or other third party material in this Peer Review File are included in the article's Creative Commons license, unless indicated otherwise in a credit line to the material. If material is not included in the article's Creative Commons license and your intended use is not permitted by statutory regulation or exceeds the permitted use, you will need to obtain permission directly from the copyright holder.
To view a copy of this license, visit https://creativecommons.org/licenses/by/4.0/

RESPONSE TO REVIEWERS — Original Reviews are coloured in Black

RESPONSES are coloured in light blue

NEW TEXT is coloured in Dark Blue and formatted as italics

Reviewer #1 (Comments for the Author):

Summary of the key results

This is a very interesting and valuable manuscript, as it demonstrates the practical applicability of tritium (^{3}H) released from nuclear power plants as a natural environmental tracer in Managed Aquifer Recharge (MAR) systems. The authors combine high-frequency sampling of δ^{18}O, δ^2H, deuterium excess (Dex), and ^{3}H with non-parametric time-series deconvolution to reconstruct groundwater travel times in a large MAR system along the Rhine River. The results show that tritium released from NPPs provides a clear, quasi-conservative tracer signal, while Dex reflects seasonal meltwater dynamics and also functions as an effective tracer. The derived travel-time metrics support improved delineation of wellhead protection zones and optimization of MAR operational strategies.

Originality and significance

The work is significant and timely. Although the use of environmental tracers in MAR studies is well established, the explicit demonstration that routine tritium releases from nuclear power plants can be used as operational tracers is novel and highly relevant.

Data & methodology

The dataset is extensive, high-resolution, and carefully collected. The methodology—particularly the use of non-parametric deconvolution—is appropriate and clearly described. The high temporal resolution of the stable isotopes and ^{3}H is a major strength.

The figures are convincing:

Figure 1: There is a data gap for tritium at Wesel between 2017 and 2020. WISER refers to the publication by Schmidt et al., which includes at least the data for 2018. I recommend incorporating these data and citing the publication accordingly.

We thank the reviewer for highlighting this and would have liked to include the respective data. We have tried to access the data described in Schmidt et al. on the indicated governmental ftp server but, unfortunately, the server access as indicated in Schmidt et al is not working anymore (apparently the ftp user password is not valid anymore). Therefore, we cannot not include this data in our figure. We include a reference to the dataset and a note that additional data appears to exist for the station Weser along the Rhine, but at the same time hope that the data will be made available either on WISER (ideal) or a FAIR data repository in the near future.

Line 330 ff: The ability to measure tritium directly at such low concentrations is remarkable. The manuscript would benefit from a more detailed description of the analytical method and additional references, especially because the Aloka LSC_LBIII instrument may not be widely known within the tritium/LSC community.

We have expanded the methods section to include a more detailed description of the analytical method and included 3 references of other papers that outline the method, precision and uncertainties in even greater detail.

Revised text (lines 425-448 of the revised manuscript): "*Similarly, careful procedures were applied for the collection, analysis, and reporting of tritium (^{3}H) activities. Tritium concentrations in water samples were determined by low-level liquid scintillation counting (LSC) at the University of Bern, Switzerland. Measurements were performed using a large-volume, ultra-low-background liquid scintillation spectrometer (Aloka LSC-LBIII) equipped with active and passive background reduction. Additional background suppression is achieved by housing the instrument in the University of Bern's underground laboratory, located approximately 46 m below the surface in sandstone bedrock, corresponding to ~75 m water-equivalent shielding. This reduces the cosmic-ray–induced muon flux by a factor of ~11*[74].

For each measurement, approximately 70 g of water were mixed with 60 g of Ultima Gold scintillator in 150 mL vials. After dark storage for about one week to allow stabilization, each vial was measured repeatedly (six to ten counting intervals of 100 min each), resulting in total counting times of roughly one week per sample. Beta decay events were recorded using a multichannel analyzer over a linear energy range of 0–180 keV, with tritium evaluated within the characteristic energy window of its 18.6 keV beta spectrum. Instrument calibration was carried out using internal tritium standards covering the relevant activity range, and additional reference standards (0–100 TU) were regularly measured to monitor counting efficiency and background stability. This approach yields a detection limit of 2–4 TU without the need for electrolytic enrichment. Measurement uncertainty was derived from counting statistics and variability among repeated measurements, yielding typical uncertainties of approximately ±1.8 TU (1σ) for the sample masses and counting times applied here. Measured count rates were corrected for radioactive decay between sampling and analysis, and background contributions were quantified using blank samples. [75]*The analytical performance of the Aloka LSC LBIII system and the applied calibration and correction procedures have been independently validated in interlaboratory proficiency tests and documented in previous methodological studies* [48,52,76]."

Appropriate use of statistics and treatment of uncertainties

The statistical treatment appears appropriate, particularly the use of Monte Carlo realizations to quantify uncertainty in the transfer functions. The discussion of travel-time variability and the limitations associated with binning is transparent and well reasoned.

Conclusions: robustness, validity, reliability

The conclusions are well supported by the presented results. The agreement between the transfer functions derived from Dex and ^{3}H increases confidence in the robustness of the approach.

We acknowledge the reviewer's positive assessment of the mathematical approaches and conclusions and left these respective aspects unchanged.

Suggested improvements

To further strengthen the manuscript, the following additions could be considered:

Clarification of the U-shaped tritium pattern in Figure 3 (for the abstracted water). Are older time series from the observation well available to help interpret this feature?

Regarding Figure 3, we now explicitly explain the observed U-shaped pattern in abstracted ^{3}H concentrations as the combined effect of (i) temporally variable NPP release signals, (ii) dispersion and mixing along flow paths, and (iii) persistence of previously infiltrated, tritium-enriched water during periods of low or absent upstream releases. We clarify that this feature does not indicate radioactive decay dominance but rather reflects the convolution of multiple release events with the system's residence time distribution. Unfortunately, there are no older time series, from the monitored abstraction well, to assess if this trend repeats annually, although the regularity of annual ^{3}H releases as visible in the riverine ^{3}H concentration records do suggest that the pattern existed throughout the last few decades.

Revised text (lines 191-197 of the revised manuscript): "*The resulting U-shaped pattern in abstracted ^{3}H concentrations reflects the convolution of temporally variable NPP release signals with the system's residence time distribution, rather than dominance of radioactive decay or seasonality in atmospheric background ^{3}H concentrations. It arises from the combined effects of temporal variability high NPP ^{3}H inputs, the dispersion and mixing of enriched infiltrate waters (supported by the $\delta^{18}O$ and $\delta^{2}H$ record) and the persistence of NPP-produced ^{3}H in abstracted waters during periods where NPP waters are not present in the infiltrated water.*"

A more explicit discussion of how MAR system geometry (e.g., hydraulic gradient, variability in infiltration rates) may influence the deconvolution results.

We have added a paragraph discussing how the site specific but also generic MAR system geometry influences the derived transfer functions. Using site-specific hydraulic gradients, infiltration rates, and known heterogeneity, we explain how these factors shape the width, skewness, and truncation of the site specific travel-time distribution. By clarifying how increased spatial heterogeneity and more variable infiltration and abstraction rates (i.e. more variable hydraulic gradients) would broaden the transfer function, we highlight how the deconvolution approach is able to inherently capture geometric variability as a probability distribution, thereby making the resulting travel-time metrics robust to geometric complexity and highly transferable.

Revised text (lines 277-285 of the revised manuscript): "*Systems with morphologies and forcings that differ from the Hardwald MAR scheme, which is characterized by constant infiltration and abstraction rates, are expected to yield systematically different deconvolution responses. Specifically, (i) higher hydraulic gradients would result in narrower transfer function distributions, (ii) structural heterogeneity would introduce skewness reflecting the coexistence of fast and slow flow pathways, and (iii) spatially distributed infiltration zones would modify the apparent truncation times of the inferred transfer functions. More generally, the ability of deconvolution methods to resolve transient groundwater recharge, flow, and discharge processes is constrained by the distinctness of the input signals, the persistence of tracers along flow paths, and the extent of mixing and dispersion within the system.*"

References

The reference list is extensive and appropriate. However:

Citation 56 contains a formatting error and should be corrected.

Corrected in the revised manuscript.

Clarity and context

The manuscript is generally well written, and the abstract is clear and informative. The introduction presents the problem and novelty of the study in a coherent manner. The results section is logically structured and supported by well-designed figures. The conclusions are clear and consistent with the data.

We thank the reviewer for the positive assessment.

Reviewer #2 (Comments for the Author):

The manuscript presents an interesting example of utilizing 3H in addition to water stable isotopes to infer groundwater travel times in a managed aquifer recharge system associated with the presence of effluents of nuclear power plants (NPP). The authors successfully managed to demonstrate the usability of 3H as travel time tracer where

increases of 3H are present due to water discharges related to NPP, replicated also by the d-excess due to the seasonal meltwater signal.

I don't have any significant comments, apart for one concern on the relatively short observation period of 1 year for the identification of seasonal trends (e.g., in terms of d-excess), exemplified by the weak correlation in the validation test, and some minor technical remarks indicated in the annotated version attached.

We thank the reviewer for the positive assessment and acknowledge the mentioned limitation of the observation period duration more clearly in the revised manuscript. We clarify that while multi-year records would strengthen statistical confidence, the d-excess signal in this system is driven by strong, repeatable seasonal snowmelt dynamics, which produce a sufficiently coherent signal even over a single annual cycle. We also emphasize that d-excess is used here in conjunction with ^{3}H, and that the convergence of independent tracers strengthens confidence in the derived travel-time distributions despite the limited temporal window.

I think this case study is a nice contribution and recommend acceptance after applying the minor corrections indicated.

Responses to technical remarks enclosed in PDF of Reviewer 2:

Line 31: the deuterium excess parameter is commonly denoted as d not Dex (see https://doi.org/10.1111/j.2153-3490.1964.tb00181.x; https://doi.org/10.25844/h9q1-0p82). Please modify accordingly throughout the text.

We agree and now consistently use "d-excess" throughout the manuscript and define it explicitly upon first use.

Line 52: first mention in the main text, expand to "managed aquifer recharge (MAR)"

This line has been amended to define MAR appropriately for the first time in text.

Line 222: unclear wording, please rephrase.

The sentence has been amended to improve clarity and grammatical correctness.

Amended sentence (lines 239-241 of the revised manuscript): "*The longer tailings (or truncation time) of the travel time transfer functions of d-excess and ^{3}H, indicate that a large portion of the infiltrated water can remain in the system for weeks to months (36.7-58.8 days).*"

All additional minor technical remarks provided in the annotated pdf have been implemented in the revised version.
